# Stability and Release Kinetics of an Advanced Gliclazide-Cholic Acid Formulation: The Use of Artificial-Cell Microencapsulation in Slow Release Targeted Oral Delivery of Antidiabetics

**DOI:** 10.1007/s12247-014-9182-5

**Published:** 2014-04-09

**Authors:** Armin Mooranian, Rebecca Negrulj, Sangeetha Mathavan, Jorge Martinez, Jessica Sciarretta, Nigel Chen-Tan, TK Mukkur, Momir Mikov, Mladena Lalic-Popovic, Maja Stojančević, Svetlana Golocorbin-Kon, Hani Al-Salami

**Affiliations:** 1 Biotechnology and Drug Development Research Laboratory, Curtin Health Innovation Research Institute, Biosciences Research Precinct, Curtin University, Perth, WA Australia; 2 Faculty of Science & Engineering, Curtin University, Perth, WA Australia; 3 Curtin Health Innovation Research Institute, Biosciences Research Precinct, School of Biomedical Science, Curtin University, Perth, WA Australia; 4 Department of Pharmacology, Toxicology and Clinical Pharmacology, Faculty of Medicine, University of Novi Sad, Novi Sad, Serbia; 5 Department of Pharmacy, Faculty of Medicine, University of Novi Sad, Novi Sad, Serbia; 6 Faculty of Pharmacy, University of Montenegro Podgorica, 8100 Podgorica, Montenegro

**Keywords:** Artificial-cell microencapsulation, Diabetes, Bile acid, Gliclazide

## Abstract

**Introduction:**

In previous studies carried out in our laboratory, a bile acid (BA) formulation exerted a hypoglycaemic effect in a rat model of type-1 diabetes (T1D). When the antidiabetic drug gliclazide (G) was added to the bile acid, it augmented the hypoglycaemic effect. In a recent study, we designed a new formulation of gliclazide-cholic acid (G-CA), with good structural properties, excipient compatibility and exhibits pseudoplastic-thixotropic characteristics. The aim of this study is to test the slow release and pH-controlled properties of this new formulation. The aim is also to examine the effect of CA on G release kinetics at various pH values and different temperatures.

**Method:**

Microencapsulation was carried out using our Buchi-based microencapsulating system developed in our laboratory. Using sodium alginate (SA) polymer, both formulations were prepared: G-SA (control) and G-CA-SA (test) at a constant ratio (1:3:30), respectively. Microcapsules were examined for efficiency, size, release kinetics, stability and swelling studies at pH 1.5, pH 3, pH 7.4 and pH 7.8 and temperatures of 20 and 30 °C.

**Results:**

The new formulation is further optimised by the addition of CA. CA reduced microcapsule swelling of the microcapsules at pH 7.8 and pH 3 at 30 °C and pH 3 at 20 °C, and, even though microcapsule size remains similar after CA addition, percent G release was enhanced at high pH values (pH 7.4 and pH 7.8, *p* < 0.01).

**Conclusion:**

The new formulation exhibits colon-targeted delivery and the addition of CA prolonged G release suggesting its suitability for the sustained and targeted delivery of G and CA to the lower intestine.

## Introduction

Diabetes mellitus is a metabolic disorder classified as type 1 (T1D) or type 2 (T2D). T1D is an autoimmune disease marked by the destruction of the β-cells of the pancreas resulting in a partial or complete lack of insulin production and the inability of the body to control glucose homeostasis [1]. T2D develops due to genetic and environmental factors that lead to tissue desensitization to insulin [2]. Despite strict glycaemic control and the fact that new and more effective antidiabetic drugs are continuously appearing in the market, diabetic patients still suffer from the disease and its complications. Antidiabetic drugs are effective in minimizing variations between peaks and troughs of blood glucose levels in diabetic patients. Common antidiabetic drugs include sulphonylureas, such as glipizid that enhances insulin production and improve insulin sensitivity, and bigvanides such as metformin which reduces glucose production in the liver and low-density lipoprotein. However, the risks of hypoglycaemia and toxin build up in the gut remain as major issues associated with diabetes especially in the presence of compromised liver and kidney functions [3]. Some drugs used for T2D, such as gliclazide (G), have beneficial extrapancreatic effects that include antiplatelet, antiradical and antioxidants effects, so some T2D patients continue to use gliclazide (G) after the loss of insulin secretion since it provides better glycaemic control than insulin alone [4–6]. In addition, the slow release kinetics of G formulation is favourable in order to prevent a sudden increase in blood glucose level after food intake. Thus, despite the availability of slow release G formulations, its PK and PD parameters in diabetic patients remain variable [7, 8].

In previously published work, significant changes in the gut segmental pH and the composition and amount of luminal bile acids (BAs) have been shown to alter the oral absorption characteristics of G [9, 10]. BAs are naturally produced in humans and, recently, have shown great potential in the treatment of diabetes [10]. Recent studies in our laboratory demonstrate significant hypoglycaemic and antidiabetic activities of G-BA formulations [11–13]. In order to design a novel and stable oral delivery system for the targeted delivery of G and optimise its efficacy through the incorporation with BAs, a suitable polymer-based matrix is needed [14, 15].

Sodium alginate (SA) is a biodegradable polymer with desirable biocompatible, biodegradable, hydrophilic and protective properties and have shown great stability, compatibility and delivery potentials in various targeted delivery slow release formulations [16, 17]. The properties of the alginate vary from one species to another and it depends on its origin [18]. Accordingly, the variability in its physical characteristics, being a natural polymer, in terms of viscosity, molecular weight, biocompatibility and other properties, can impact its functionality in a formulation and drug release profile. This is anticipated to have an impact on our formulation and has been thoroughly investigated to select a specific alginate polymer for best outcome in terms of stability and release studies [19].

SA has well-established pH-release kinetics and is significantly influenced by the viscosity of its matrix [20]. Recent studies have demonstrated a faster and well-controlled drug release from the low-viscosity SA (LVSA) compared with high-viscosity SA (HVSA) suggesting superior performance of LVSA especially when targeting distal sites of the GIT such as the cecum [21].

pH is known to significantly influence the physicochemical properties of LVSA [22], thus, this should be examined when designing a new G-CA-SA formulation. In gastric fluid, the hydrated LVSA is converted into a porous, insoluble acid matrix. Theoretically, alginate shrinks at low pH and the encapsulated G and CA are not released. Once passed into higher pH of the GIT, the alginic acid matrix is converted to a soluble viscous layer. In our recently published work, a basic formulation of G-CA-SA was absorbed poorly from the ileum of diabetic rats [11] suggesting a great potential for a new and enhanced absorption when targeting the cecum using LVSA. Since LVSA is substantially influenced by the change in pH [22], the newly designed formulation should be tested for drug release at various pH values. We have also shown that various formulations of bile acids and G have great potential in the treatment of T1D [9, 11]. Accordingly, a successful oral delivery system needs to be developed which consists of G formulated in a pH-controlled LVSA biodegradable polymer mixed with the bile acid, CA, using artificial-cell microencapsulation (ACM) technology (Buchi-based microencapsulating system) which we optimised in our laboratory. Parameters were set in frequency range of 1,000–1,500 and a flow rate of 4 ml/min under consistent air pressure of 1.5 bar. A newly designed formulation of LVSA microcapsules containing G and the CA has been recently produced and characterised (unpublished data). These new microcapsules exhibit good microcapsule morphology and rheological parameters suitable for oral delivery. Accordingly, this study aims at characterising this formulation and examining the effect of CA on G release kinetics at various pH and temperature values.

## Materials and Methods

### Materials

Gliclazide (99.92 %), low-viscosity sodium alginate (99 %) and cholic acid (98 %) were purchased from Sigma Chemical Co, USA. Calcium chloride dehydrate (98 %) was obtained from Scharlab S.L, Australia. All solvents and reagents were supplied by Merck (Australia) and were of HPLC grade and used without further purification.

### Drugs Preparations

Stock suspensions of G (20 mg/ml) and CA (1 mg/ml) were prepared by adding the powder to 10 % ultra water-soluble gel. The CaCl_2_ stock solution (2 %) was prepared by adding CaCl_2_ powder to HPLC water. All preparations were mixed thoroughly at room temperature, for 4 h, stored in the refrigerator and used within 48 h of preparation.

### Preparation of Microcapsules

Microcapsules of G-loaded low-viscosity sodium alginate (LVSA) were prepared using our Buchi-390-based microencapsulating system. Polymer solutions containing SA and G with or without CA were made up to a final concentration (of G-CA-SA) in a ratio of 1:3:30, respectively [9, 11]. Microcapsules were collected from our microencapsulating system, and for each formulation, three independent batches were prepared and tested separately (*n* = 3). All microcapsules (unloaded microcapsules, G-loaded microcapsules and G-CA-SA-loaded microcapsules) were prepared and treated in the exact same way. Microcapsules were dried by using the stability chambers (Angelantoni Environmental and Climatic Test Chamber, Italy). The weight of the recovered dry particles was recorded, and the G contents, production yield, the microencapsulation efficiency, zeta potentials and mean particle size of each preparation were all measured and compared, as described below.

### Characterization of Loaded Microcapsules

#### Drug Content, Production Yield, Microencapsulation Efficiency and Stability Studies

##### Drug Content, Production Yield and Microencapsulation Efficiency

One gram of microcapsules were carefully weighed, ground and dissolved in 200 ml of phosphate buffer (pH 7.8), and the suspension was stirred by a magnetic stirrer for 6 h. Two millilitres of the solution was transferred to a 100 ml flask and diluted with phosphate buffer (vehicle) to 100 ml. Aliquots of the dissolution medium (2 ml) were withdrawn at predetermined time points (every 200 s) and filtered through Millipore, 0.22 μm filter. Amount of dissolved drug was determined spectrophotometrically at 229 nm against the buffer as blank [23, 24]. The measurements were performed under sink conditions, and average values were calculated. Validated method for UV-G analysis was developed [25] for both formulations, G-SA and G-CA-SA, and method’s accuracy and precision was confirmed using previously published HPLC methods [11]. Absorbances were measured using UV spectrophotometer (Shimatzu UV–Vis spectrophotometer 1240, Japan). G concentrations were calculated from the calibration curve. All analyses were carried out in triplicate (*n* = 3). We calculated the drug contents, production yield and microencapsulation efficiency from the following equations [26]:

1 $$ \%\mathrm{Drug}\ \mathrm{content}=\frac{\mathrm{Calculated}\kern0.5em \mathrm{amount}\kern0.5em \mathrm{of}\kern0.5em \mathrm{G}\kern0.5em \mathrm{in}\kern0.5em \mathrm{the}\kern0.5em \mathrm{microcapsules}}{\mathrm{Total}\kern0.5em \mathrm{weight}\kern0.5em \mathrm{of}\kern0.5em \mathrm{microcapsules}}\times 100 $$

2 $$ \%\mathrm{Production}\kern0.5em \mathrm{yield}=\frac{\mathrm{Total}\kern0.5em \mathrm{weight}\kern0.5em \mathrm{of}\kern0.5em \mathrm{the}\kern0.5em \mathrm{microcapsules}}{\mathrm{Total}\kern0.5em \mathrm{weight}\kern0.5em \mathrm{of}\kern0.5em \mathrm{the}\kern0.5em \mathrm{polymers}}\times 100 $$

3 $$ \%\mathrm{Encapsulation}\kern0.5em \mathrm{efficiency}=\frac{\mathrm{Drug}\kern0.5em \mathrm{content}}{\mathrm{Theoretical}\kern0.5em \mathrm{content}}\times 100 $$

##### Physical and Chemical Stability

The stability test was carried out by placing predetermined amounts of freshly prepared microcapsules onto sterile petri dishes (30 microcapsules in each) and storing them in thermostatically controlled ovens at −20, 5, 25 and 40 °C with relative humidity set at 35 % for 3 days. The experiment was conducted using stability chamber as described above. A temperature and humidity regulator was used to ensure constant experimental conditions. At the end of the experiment, the microcapsules were analysed for any changes in appearance and for the determination of the amount of drug remaining in each formula using a validated UV–Vis stability-indicating method. Briefly, the dosage forms were crushed and dissolved in 200 ml phosphate buffer at pH 7.8. The solution was filtered and the first 20 ml of the solution was removed, and 10 ml of the filtrate was diluted to 100 ml in a volumetric flask. One-millilitre aliquot of the prepared solution was transferred to a 10-ml volumetric flask, and the volume was completed with the buffer. A calibration curve was constructed for G in phosphate buffer across the concentration range of 0.1 to 40 mg/ml with *R* ^2^ = 0.99 (data not shown).

##### Zeta Potential and Size Analysis

To determine the electrokinetic stability and size uniformity of the microcapsules in the colloidal system, zeta potential and size distribution for the microencapsulated formulations of G-SA and for G-CA-SA were measured by photon correlation spectroscopy using a Zetasizer 3000HS (Malvern Instruments, Malvern, UK) and by Mie and Fraunhofer scattering technique using Mastersizer 2000 (Malvern Instruments, Malvern, UK). The measurements were performed at 25 °C with a detection angle of 90^°^, and the raw data were subsequently correlated to *Z* average mean size using a cumulative analysis via OmniSEC-Zetasizer software package. Each sample was measured 10 times. All analyses were performed on samples appropriately diluted with filtered deionized water. All determinations were performed in triplicate. Results are reported as mean ± SD.

#### Drug Release Studies (In Vitro Dissolution Test)

A weighed sample (2 g) of G- and CA-loaded microcapsules were suspended in 200 ml of phosphate-buffered solution at pH values of 1.5, 3, 7.4, and 7.8 for 2 h, as appropriate. The dissolution medium was stirred at 200 rpm. Sink conditions were maintained throughout the assay period. All the experiments were carried out at 25 °C. All absorbances of the solution were measured every 200 s using our Hewlett-Packard-based time-controlled UV-Spec mounted with close-loop flow system. All analyses were carried out in triplicate (*n* = 3).

#### Swelling Studies

To determine the swelling properties of the microcapsules, 50 mg dry microcapsules were weighed and placed in 20 ml of two pH values (3 and 7.8) and two temperatures (20 and 30 °C) for 6 h. The choice of two temperature points were based on previously published work and a 10° difference needed for comparison [27, 28]. The swollen microcapsules were then removed at periodically predetermined intervals (hourly). The wet weight of the swollen microcapsules was determined by blotting them with filter paper to remove moisture adhering to the surface, immediately followed by weighing on an electronic balance. All experiments were done in triplicate. The percentage of swelling of the microcapsules was calculated from the following formula [26]:

4 $$ \mathrm{Weight}\kern0.5em \mathrm{ratio}=\frac{\mathrm{Final}\kern0.5em \mathrm{weight}}{\mathrm{Initial}\kern0.5em \mathrm{weight}}\times 100 $$

#### Statistical Analysis

Values are expressed as means ± SD. Drug content, production yield and microencapsulation efficiency were assessed using Student’s *t* test. Statistical comparisons between gliclazide concentrations in different microencapsulated formulations were carried out by repeated measures analysis of variance (ANOVA) using each formulation excipients as fixed terms. Swelling studies were assessed using two-way ANOVA to assess the main effects of microcapsule formulation and pH and their two-way interaction. Tukey HSD post hoc comparison of means was done only when the associated main effect or interaction was statistically significant. The best fit model was derived using GraphPad Prism software (v6; GraphPad Software, Inc., USA). Statistical significance was set at *p* < 0.05. For all statistical analysis, the program SPSS (IBM SPSS, version 20, USA) was used.

## Results and Discussion

### Drug Content, Production Yield, Microencapsulation Efficiency and Stability Studies

As shown in Table 1, the results for percentage drug content of initially added drug for both formulations revealed consistent drug-microcapsule content with very low variations. As expected, there is less G in the G-CA-SA microcapsules compared with G-SA microcapsules (*p* = 0.04). The total production yield of G-SA and G-CA-SA microcapsules prepared ranged from 78 to 90 % with no significant difference between both formulations. Good levels of G-loading (microencapsulation) efficiency were achieved for all microcapsules, with values averaging 90 ± 10 %.

**Table 1:** Drug contents, production yield, encapsulation efficiency, zeta potential and mean particle size (*n* = 3)

Formulation code	Formula composition	Drug content (%) ± SD	Production yield (%) ± SD	Encapsulation efficiency (%) ± SD	Zeta potential (mV) ± SD	Mean particle size (μm) ± SD
G-SA	Gliclazide-low-viscosity sodium alginate microcapsules	5 ± 0.2	83 ± 5	95 ± 7	−66 ± 1.6	900 ± 1
G-CA-SA	Gliclazide-cholic acid-low-viscosity sodium alginate microcapsules	3 ± 0.1^*^	84 ± 6	83 ± 6	−46 ± 1	938 ± 1

^*^ *p* < 0.05

#### Stability, Zeta Potential and Size Analysis

Accelerated stability studies were carried out over a 3-day period, testing both formulations at −20, 5, 25 and 40 °C and relative humidity at 35 %. Upon visual examination, both formulations (G-SA and G-CA-SA) retained their original morphological characteristics (sphericity and homogenous particle size distribution) across the experimental conditions. However, there were some changes in the colour, overall size and quality of the microcapsule surfaces at higher temperature. Specifically, at −20 °C, microcapsules retained their original size and some had formed agglomerates which were easily re-dispersed. At this temperature, microcapsules were white and spherical and had retained their original quality (soft and flexible). At the higher temperatures, the microcapsules changed colour due to oxidation of the alginate from cream to orange at 5–25 °C and brown at 40 °C, and whilst retaining their spherical changes and even homogenous particle size distribution, they had shrunk in size by 50 %, with the smallest microcapsules being at the highest temperature of 40 °C. This may be explained by the loss of moisture content, reducing the overall surface area and volume of each microcapsule. In addition, the microcapsules at all temperatures (except at −20 °C) had become harder and more brittle owing to loss of moisture within the microcapsules.

Upon UV analysis, the amount of G remaining in freshly prepared microcapsules revealed an average percentage drug content compared with initially added drug of five for G-SA microcapsules and three for G-CA-SA (Table 1). This complemented the visual characterisation of the microcapsules following accelerated stability testing and confirmed uniformity of drug contents and is in line with the used drug: polymer ratios (G-SA 1:30 and G-CA-SA 1:3:30). Neither any peaks for a biodegradable polymer nor any alteration of the chromatographic pattern of G was observed, indicating that the experimental conditions for the microencapsulating process did not compromise drug analysis. Furthermore, the results of the drug content and encapsulation efficiency showed minimum variation among repeated samples which confirms the reproducibility and of our developed microencapsulation method.

The dispersion of microcapsules is stable as shown by zeta potential values ranging from −45 to −68 mV, whilst the mean particle size remained within a narrow range of 900–939 μm suggesting significant uniformity in the size distribution of the microcapsules and no significant difference after the incorporation of CA to G-microcapsules (Table 1).

### Drug Release Studies and In Vitro Dissolution

**Fig. 1:**
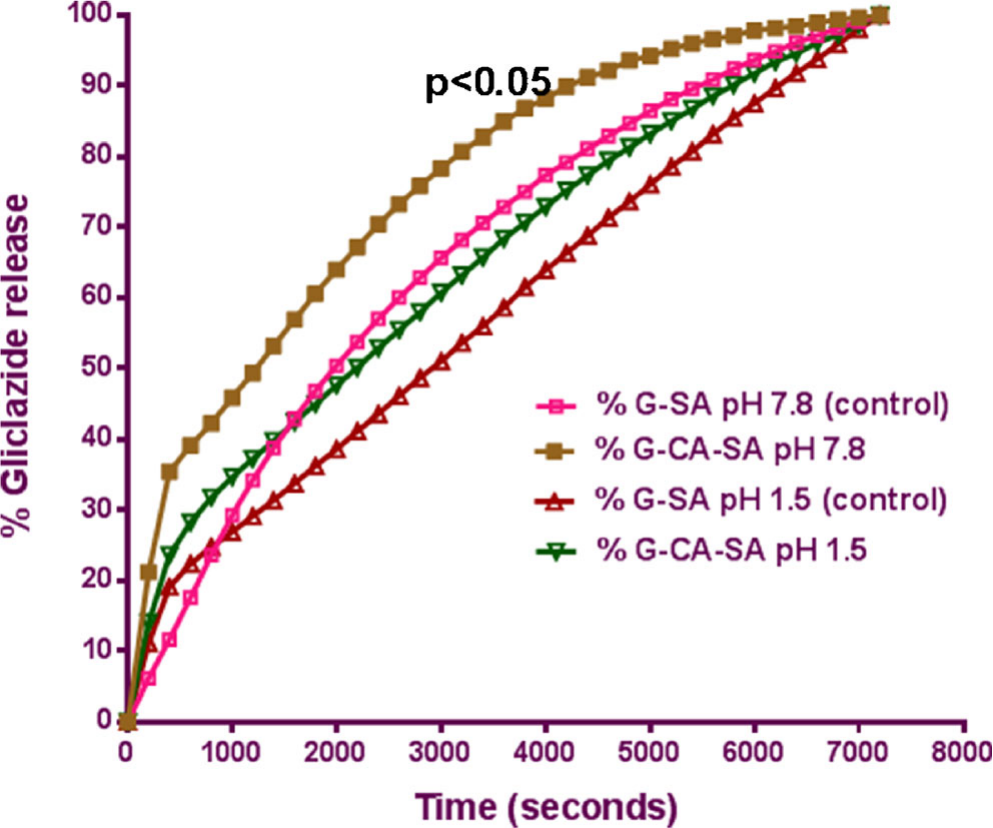
Gliclazide release over time from G-SA and G-CA-SA microcapsules at pH 1.5 and pH 7.8

**Fig. 2:**
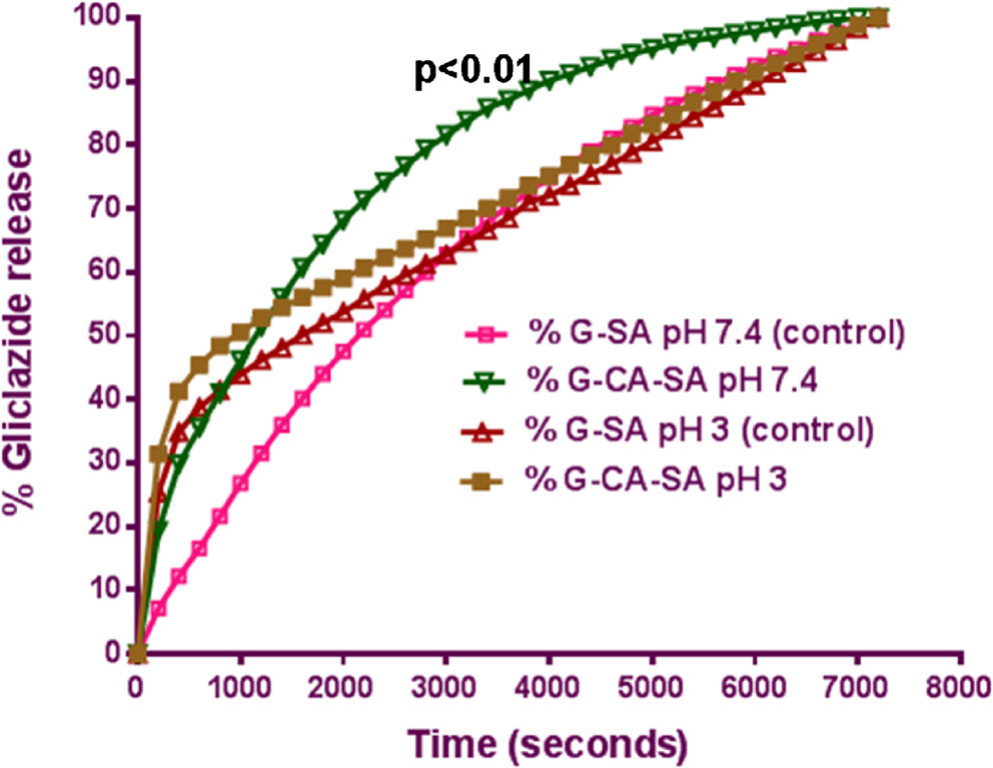
Gliclazide release over time from G-SA and G-CA-SA microcapsules at pH 3 and pH 7.4

Gliclazide release from the microcapsules was studied in triplicate across 4 pH values (1.5, 3, 7.4 and 7.8) at 20 °C using both formulations G-SA and G-CA-SA. However, it is worth stating that, another approach would have been to use gradient pH medium which will also give improved findings. pH values were chosen based on our previous studies examining best sites of drug absorption in the GIT [9, 11, 13, 29–32]. The release of G was slower and largely dependent on both the pH and the composition of the coating. As shown in Figs. 1 and 2 and Table 2, G release was smaller at low pH values (1.5 and 3) and the bile acid coating in the G-CA-SA formulation allowed for a slower rate of drug release. As expected, the release of G was increased at higher pH values (especially at pH 7.8) for both formulations. Notably, at pH 7.4 and 7.8, *more than 80* % *of the G was released in the first hour* (*3*,*600 s*) *at pH 7.4* (Fig. 2) (*and similar or higher amounts were released at pH 7.8* (Fig. 1) *for the formula coded G*-*CA*-*SA*). This has important ramifications for diabetes therapy, as previous work in our laboratory has confirmed the distal ileum as the intended site of G absorption where pH values are in the range of 7–7.8 [9, 11, 13, 15, 30–32]. Our results demonstrate that the CA-reinforced microcapsules stipulated controlled drug release at the targeted pH of 7.8.

**Table 2:** Statistical analysis of Figs. 1 and 2

Group comparison		pH	*p* values
% G-SA pH 7.8 (control)	% G-CA-SA pH 7.8	7.8	0.042
% G-SA pH 1.5 (control)	% G-CA-SA pH 1.5	1.5	0.068
% G-SA pH 7.4 (control)	% G-CA-SA pH 7.4	7.4	<0.001
% G-SA pH 3 (control)	% G-CA-SA pH 3	3	0.79

The mechanisms responsible for such differences in the release profiles are affected by the pH of the medium. At low (stomach) pH, the hydrated SA is modified to a porous structure known as alginic acid skin [9, 11, 13, 15, 30–32]. This insoluble structure results in the shrinkage of alginate and thus encapsulated drugs are not completely released [24]. However at higher pH (intestine) such as the distal end of the small intestine, the alginic acid forms a soluble viscous layer due to the rapid dissolution and solubilisation of the alginate matrix resulting in the burst of encapsulated drugs [19, 24]. As evident from the G-SA dissolution graphs (Figs. 1 and 2), this release profile is not in a sustained, controlled pattern and may result in reduced bioavailability of G.

Whilst there is substantial evidence regarding bile salt-reinforced microcapsules used to ensure controlled drug release from microcapsules, little is known about the incorporation of bile acids rather than bile salts in the microencapsulation formulation [24, 33–35]. In this study, the bile acid cholic acid was used. In our previous work, we used a similar formulation (without microencapsulation) and have shown an antidiabetic effect in an animal model of type-1 diabetes [9, 11, 13, 15, 30–32, 36]. We have also shown through ex vivo studies that the cholic acid derivative does enhance G permeation through the ileal mucosa of diabetic rats [9, 11, 13, 15, 30–32].

Our dissolution and release studies using microcapsules from both formulations may provide an explanation as to why the CA derivative has improved the G absorption through the ileum [9, 11, 13, 15, 30–32]. We therefore hypothesize that the bile acid, CA, reinforces the soluble polymer alginate matrix by such that rapid burst of drug is evaded at higher pH values, and this brings about a partial prolonged release targeting the lower intestine facilitating better absorption and less gut metabolism. However, due to the fact that in our previous work, we did not microencapsulate our G-CA-SA formulation, the improvement of G permeation by CA was rather limited. Additionally, unlike bile salts which contain both hydrophilic and hydrophobic ends due to their zwitterionic structure, bile acids lack good solubility and, thus, resist greater dissolution and solubilisation at higher pH values whilst retaining cross-linking (ionotropic bridging) with the alginate formulation of the microcapsules [9, 11, 13, 15, 30–32, 36–39]. It is worth stating that a limitation to our findings is that the release of the microcapsules was studied over a period of only 2 h at each particular pH value. Having said that, our results provide some explanation to our published work, where a G-bile acid mixture produced small but significant increase in the release kinetics and absorption of G in diabetic rats [11]. In addition, the results suggest that a LVSA-microencapsulated G-CA-SA formulation produced using a 1:30 ratio exhibit a slow release and optimum G delivery in pH 7.8 at 30 °C. One of the potential advantages of using bile acids over bile salts is that they are far less soluble whilst still forming cross-links with the matrix and help to stabilise the membrane from rapid disintegration, as shown in Figs. 1 and 2 and Table 2. Accordingly, this formulation is promising as a platform for antidiabetic drug delivery.

### Swelling Studies

**Fig. 3:**
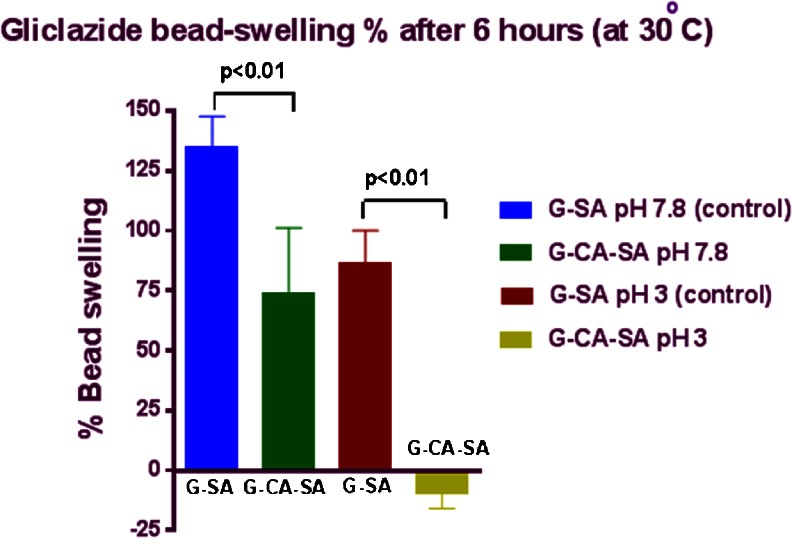
Swelling characteristics of G-SA and G-CA-SA microcapsules (pH 3 and pH 7.8) at 30 °C

**Fig. 4:**
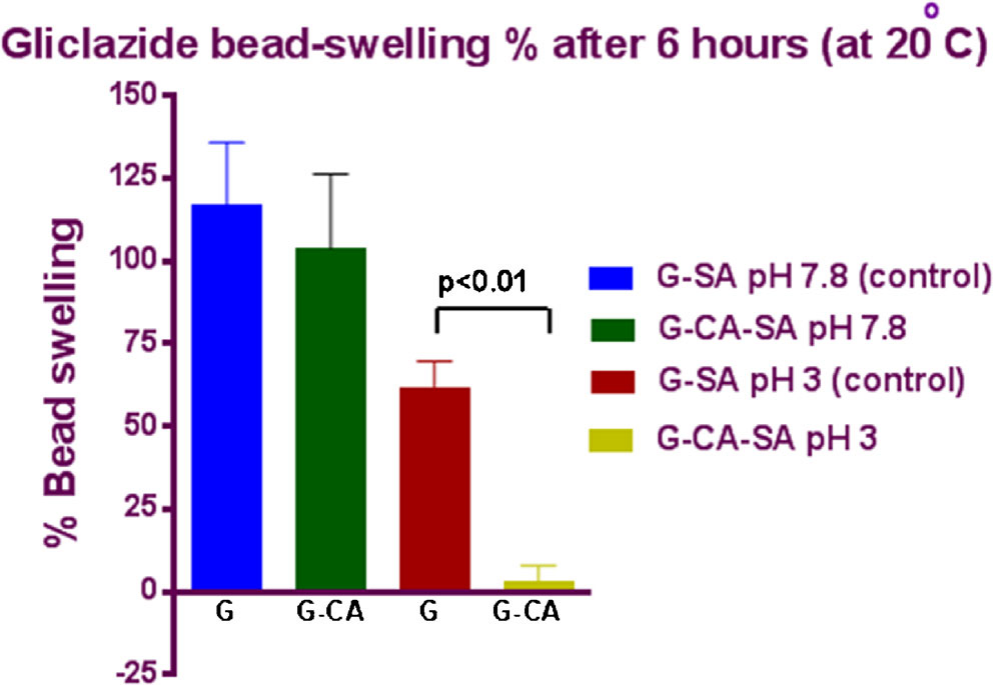
Swelling characteristics of G-SA and G-CA-SA microcapsules (pH 3 and pH 7.8) at 20 °C

Figures 3 and 4 and Table 3 show that the formulation type, pH of the medium and the temperature do have an effect on the swelling characteristics of the microcapsules. In line with G release studies, the percentage of microcapsule swelling was reduced by the addition of CA. CA reduced microcapsule swelling at low and high pH (at 30 °C, Fig. 3) and to a less extent, at lower temperature (at 20 °C, Fig. 4). CA exerted a stronger reduction in microcapsule swelling, at higher temperature suggesting better control of G release across the stomach and lower proximal site of the intestine, whilst still maintaining targeted delivery at pH 7.4 and pH 7.8 (Figs. 1 and 2). This may be due to the fact that even though alginate has been shown to undergo substantial swelling at higher pH and temperature due to higher water uptake [24, 40, 41], CA reduces such a swelling effect and thus brought about a stronger control of G release. Moreover, at high pH values, the porosity and solubilisation of the polymer matrix are expected to be also higher [19, 24, 42]. Temperature also plays a key part in swelling and water uptake as higher temperatures causes erosion and disintegration of the matrix wall allowing for greater water penetration [7, 40, 41, 43–45]. At pH 7.8 and higher temperature (30 °C), the G-SA microcapsules (without CA) experienced greater swelling than the G-CA-SA microcapsules. Again, at pH 3.0 and 30 °C, G-SA-CA microcapsules underwent greater swelling due to enhanced water uptake. This clearly supports our hypothesis that the bile acid used in the formulation has enhanced membrane stabilisation most likely via cross-linking and ionic interactions with the alginate matrix. Another possible explanation is that the free carboxyl groups of primary bile acids CA act as water binding sites allowing for greater water uptake and thus weight gain and more microcapsule-swelling capacity [9, 11, 13, 15, 30–32, 36–39]. This swelling characteristic property of bile acid-reinforced microcapsules is desirable in forming a controlled release system, especially when it does not adversely influence production yield or system stability (Table 1).

**Table 3:** Statistical analysis of Figs. 3 and 4

Group comparison		temp	pH	*p* values
G-SA pH 7.8 (control)	G-CA-SA pH 7.8	30	7.8	0.003
G-SA pH 3 (control)	G-CA-SA pH 3		3	<0.001
G-SA pH 7.8 (control)	G-CA-SA pH 7.8	20	7.8	0.931
G-SA pH 3 (control)	G-CA-SA pH 3		3	<0.001

## Conclusion

Our microencapsulation method of G and CA at the set ratio is novel for G and CA oral delivery. It produces microcapsules which display appropriate stability, release kinetics and uniformity. The formed microcapsules ensure optimal targeted delivery to the site of action (lower intestine) with enhanced stability and high consistency provided via bile acid-reinforced microcapsules. An interesting future investigation will incorporate a less polar bile acid, such as deoxycholic acid, to optimise further the release kinetics and efficacy of the antidiabetic drug, gliclazide.
